# A Case Report of Wünderlich Syndrome Causing Massive Hemorrhage During Hemodialysis

**DOI:** 10.5811/cpcem.1891

**Published:** 2024-05-14

**Authors:** Karalee Bluhm, Ravali Kundeti, Nicole Maguire

**Affiliations:** Rutgers-RWJBH- Community Medical Center, Department of Emergency Medicine, Toms River, New Jersey

**Keywords:** *Wünderlich syndrome*, *hemorrhagic shock*, *renal emergency*, *case report*, *dialysis*

## Abstract

**Introduction:**

Wünderlich syndrome (WS) refers to subcapsular, perirenal, or pararenal hemorrhage due to non-traumatic and iatrogenic conditions. Neoplasms, vascular disease, renal etiology, and anticoagulant use are underlying risk factors.

**Case Report:**

We describe a case of WS in a 79-year-old male who was undergoing hemodialysis, which resulted in hemorrhagic shock requiring multiple transfusions and embolization by interventional radiology.

**Conclusion:**

Most commonly, patients present with flank pain; a computed tomography with contrast of the abdomen is essential for diagnosis. Surgical intervention is considered in hemodynamically unstable patients. Conservative therapy and intravenous resuscitations with blood products are considered a priority in hemodynamically stable patients.

Population Health Research Capsule What do we already know about this clinical entity?

*In patients with risk factors, Wünderlich syndrome (WS) may present as the Lenk triad: acute flank pain, flank mass, and hypovolemic shock due to renal hemorrhage.*

What makes this presentation of disease reportable?

*Fewer than 300 reported cases of WS have been reported between 1974–2016. It has never been reported in patients actively undergoing hemodialysis.*

What is the major learning point?

*Presentation varies from vague to life-threatening. Prompt computed tomography will aid in diagnosis; treatment ranges from supportive to surgical management.*

How might this improve emergency medicine practice?

*This rare presentation of WS highlights the importance of a wide differential in those presenting with the Lenk triad.*

## INTRODUCTION

Wünderlich syndrome (WS) is a rare but life-threatening condition defined as acute-onset, spontaneous, non-traumatic renal hemorrhage into the subcapsular, perirenal, or pararenal spaces. Fewer than 300 documented cases have been reported between 1974–2016. It is characterized by the Lenk triad: acute flank pain, flank mass, and hypovolemic shock.^1^ However, the clinical manifestations can be varied and nonspecific. Presence of all three components of the triad is uncommon, occurring in only 20% of cases. Abdominal and flank pain are the most frequent symptoms, occurring in 67% of patients, followed by hematuria (40%) and hemorrhagic shock (26.5%).^2^

Approximately 60–65% of cases are related to renal neoplasms, with angiomyolipoma leading benign neoplasm causes and renal cell carcinoma the main cause of malignant neoplasms. The overall prevalence of WS as a complication of renal cell carcinoma occurs in only 0.3–1.4% of cases, whereas it is estimated to occur in 13–100% of cases of renal angiomyolipoma.^3^ Other causes include tuberous sclerosis, vascular lesions (eg, polyarteritis nodosa), arteriovenous malformations, renal artery aneurysms, ruptured renal cysts, renal calculi, and coagulopathy.^4^ Wünderlich syndrome is often associated with hypertension (33–50%) and atherosclerosis (80–87%).^3^ Wünderlich syndrome has a slight male preponderance with an average age of 46.8 years, with most cases occurring between 30–60 years.^2^

Traditionally, the management has ranged from symptomatic care and observation to partial or complete nephrectomy. Currently, the treatment of choice includes stabilization and transcatheter arterial embolization (TAE), but this depends on the severity of presentation or underlying cause of WS. Surgical treatment or curative nephrotomy is preferred in patients with diagnosed renal malignancy, in cases of hemodynamic instability, and/or failed TAE.^4^ ^,^ ^5^

We present the case of a 79-year-old male who presented with a spontaneous left renal subcapsular hematoma and an active perinephric hemorrhage that occurred during hemodialysis, leading to hemorrhagic shock requiring multiple blood transfusions and arterial embolization.

## CASE REPORT

A 79-year-old male presented to the emergency department with 30 minutes of acute and worsening abdominal pain that occurred suddenly during the first hour of his scheduled hemodialysis. He described the pain as sharp, cramping, stabbing, constant, and diffuse but worse in the left upper quadrant and flank and associated with acute abdominal distention. He denied any recent trauma, chest pain, shortness of breath, paresthesias, nausea, vomiting, or urinary complaints.

His past medical history included end-stage renal disease (on hemodialysis), type 2 diabetes mellitus, chronic obstructive pulmonary disease, hypertension, hyperlipidemia, and hypothyroidism. On chart review it was noted that there was a history of left renal cyst, last measuring 6.8 centimeters (cm) × 5.5 cm × 5.3 cm four years prior to presentation found incidentally on computed tomography (CT). Follow-up renal ultrasound was ordered but never obtained. The patient’s chronic kidney disease was related to his diabetes and poorly controlled hypertension that required treatment with four antihypertensive medications. His surgical history included two unspecified hernia repairs, bilateral knee replacements, loop recorder placement, and small bowel resection due to small bowel obstruction 15 years previously. His social history included daily alcohol use, and he was a non-smoker who lived with family and was retired.

Initial vital signs revealed slight hypotension with blood pressure of 98/42 millimeters of mercury; otherwise, vitals were within normal limits. The patient had been awake and alert, in obvious distress due to pain, and having difficulty sitting still. Abdominal exam was notable for being obese with rigidity, distention, diffuse tenderness, guarding, and without appreciable bowel sounds, rebound tenderness, or ecchymosis. The remainder of his exam was unremarkable.

**Image 1.**
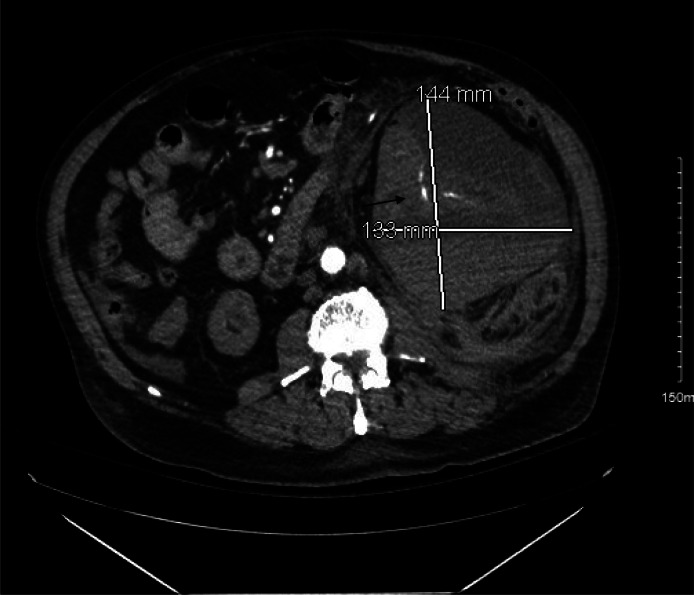
Axial view of computed tomography angiogram of the abdomen showing large left renal hematoma with contrast blush (arrow) suggestive of active bleeding. *mm*, millimeter.

**Image 2.**
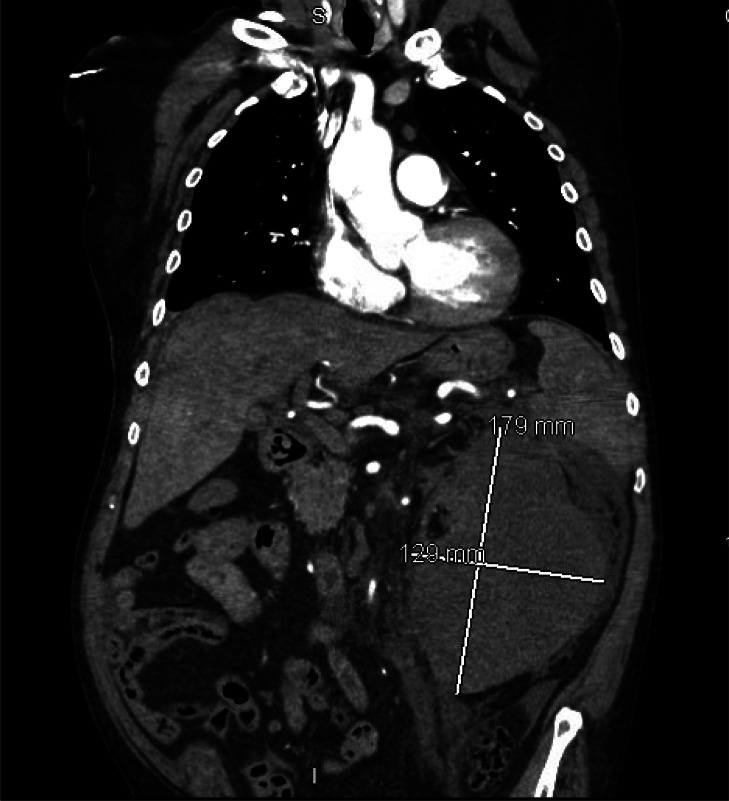
Coronal view of computed tomography angiogram of the abdomen and pelvis showing large left renal subcapsular hematoma and hemorrhage. *mm*, millimeter.

A point-of-care ultrasound (POCUS) revealed a grossly positive focused assessment with sonography for trauma (FAST) in Morrison’s pouch and splenorenal recess, with limited subxiphoid and suprapubic views. He was treated with fentanyl intravenously and bolused with normal saline. The CT angiogram of the abdomen and pelvis was notable for an 18-cm left renal subcapsular hematoma and hemorrhage in the left perinephric space with active contrast blush coming from the left renal segmental and interlobar branches (Images 1 and 2). There were also multiple sclerotic lesions of the thoracic and lumbar spine indicating possible metastasis. Pertinent lab values included white blood cell count of 14.6 × 10^3^/microliter (μL) (reference range 4.3–12.0 × 10^3^/μL), with 83% neutrophils (5–13%), hemoglobin 7.8 grams per deciliter (g/dL) (13.4–17.4 g/dL), hematocrit 24.1% (40–54%), platelets 271 × 10^3^/μL (150–440 × 10^3^/μL), international normalized ratio 1.09 (0.83–111), partial thromboplastin time 29.3 seconds (21.5–31.9 seconds), prothrombin time 12.7 seconds (10–13 seconds), point-of-care lactic acid 2.4 millimoles per liter (mmol/L) (0.0–2.0 mmol/L), glucose 204 milligrams per deciliter (mg/dL) (60–110 mg/dL), blood urea nitrogen 47 mg/dL (8–25 mg/dL), and creatinine 7.13 mg/dL (0.5–1.12 mg/dL).

While awaiting interventional radiology for renal embolization, the patient clinically deteriorated requiring emergent central line placement and two units of uncross-matched blood administered by pressure infuser and warmer for hypotension and active bleeding. During embolization, multiple branches of the left kidney including upper pole, mid-pole and lower pole subsegmental arteries, as well as interlobular and arcuate arteries at all three junctures, were selected and embolized with good angiographic result and no evidence of extravasation at the termination of the procedure. The patient was then admitted to the surgical intensive care unit for monitoring. He maintained stable blood pressures and was discharged home after four nights in the hospital.

After discharge, he was sent for hematology and oncology referral and positron emission tomography due to incidental findings on his CT during the current admission. However, the patient returned three days after discharge with hypoglycemia, severe anemia, and active upper gastrointestinal bleed. He underwent an esophagogastroduodenoscopy and exploratory laparotomy but ultimately died due to complications of the gastrointestinal bleed within three days of his subsequent admission. There was no further oncological workup performed.

## DISCUSSION

Wünderlich syndrome is a rare condition, but its occurrence in a dialyzed patient is extremely rare with only a handful of other reports in the literature.^5^ ^,^ ^6^ Wünderlich syndrome occurring during hemodialysis has never previously been reported. Our case is an uncommon example of WS as it occurred in a patient with end-stage renal disease during dialysis without a known history of malignancy that resulted in significant hemodynamic instability requiring emergent transfusion, embolization, and intensive care admission. Computed tomography with contrast or CT angiogram are the diagnostic tests of choice,^7^ but POCUS is useful in identifying intra-abdominal fluid, which may be hemorrhage.

Although the images in this report do not demonstrate fluid around the liver, POCUS exam swiftly revealed a more diffuse, intra-abdominal hemorrhage as demonstrated by the positive FAST exam, indicating that the blood had escaped the retroperitoneal perinephric Gerota fascia into the peritoneal cavity. Unfortunately, we were not able to save the ultrasound images. The use of ultrasound, while not novel, did substantially reduce the time taken from commencing care to definitive treatment. Treatment is based on the patient’s condition, but stabilization and transcatheter arterial embolization are standard of care for WS causing hemorrhagic shock.^8^ ^,^ ^9^

Our patient had several risk factors that may have contributed to the development of WS. He had a history of hypertension, renal cysts, diabetic nephropathy, and atherosclerosis. Additionally, he had a daily aspirin regimen, which might have exacerbated his outcome. A previous case of bleeding into a simple renal cyst causing hemorrhagic shock without other risk factors known to WS has been reported.^8^ Hemorrhage in our patient was located in the kidney, which had the background of renal cysts, suggesting non-malignant kidney masses as a nidus for hemorrhage that is consistent with literature review.

Furthermore, it is possible that he had an undiagnosed metastatic cancer as evidenced by lesions noted incidentally on CT. Further workup was unobtainable due to clinical deterioration. The relative risk of renal cell carcinoma in hemodialysis patients was found to be 13.3 to 29-fold higher than in normal subjects, and hemodialysis patients have a risk of 2.3–3.3% of developing renal cell carcinoma.^6^ An autopsy was not performed; therefore, biopsy of the lesion was not obtained.

The patient was on daily aspirin but otherwise was not receiving anticoagulation. His subsequent admission also revealed massive bleeding; so, it is possible there was some underlying hematological component that was causing or contributing to his bleeding diathesis. Daily alcohol use also may be a risk factor for WS or the severity of presentation. However, urologists have documented that uremic patients have a bleeding tendency associated with platelet dysfunction^10^ because platelets in uremic patients have a reduced aggregating response to adenosine diphosphate, epinephrine, and collagen.^11^ Hemodialysis patients, therefore, are more likely to have more severe bleeding, and treatment should include consideration of one or a combination of the following: cryoprecipitate, desmopressin, and conjugated estrogens.^12^

Due to our patient’s sudden presentation during hemodialysis, we considered whether hemodialysis may have been a risk factor causing enough shifts in pressure to lead to spontaneous atraumatic renal hemorrhage. Very few cases of WS occur in hemodialysis patients; therefore, this subset of patients with WS risk factors has a higher likelihood of developing WS, particularly if they present with elements of the Lenk triad.

## CONCLUSION

Wünderlich syndrome is a rare but important differential to consider in atraumatic flank and abdominal pain. Mild presentations can easily be misdiagnosed as back pain, urolithiasis, or renal infections; so, keeping this process in one’s differential, especially in patients with risk factors, is important in obtaining the diagnosis. Point-of-care ultrasound, CT, and CT angiogram are paramount in identifying intraperitoneal free fluid and active hemorrhage. Although several cases of WS have been effectively managed conservatively, patients such as ours can suffer from hemorrhagic shock resulting from the syndrome.
